# Quantitative evaluation of variables to student success in a mastery learning baccalaureate nursing programme

**DOI:** 10.1002/nop2.278

**Published:** 2019-04-07

**Authors:** Marie Rolf, Margaret Kroposki, Susan Watson

**Affiliations:** ^1^ College of Nursing Roseman University of Health Sciences Henderson Nevada; ^2^ College of Doctoral Studies University of Phoenix Tempe Arizona

**Keywords:** Assessment Technologies Institute, entrance assessment, evaluation, exit assessment, mastery learning, nursing education, pre‐admission interview, predictors, remediation, standardized testing

## Abstract

**Aim:**

This study evaluated the relationship of student input and throughput variables in a mastery learning baccalaureate nursing programme to licensure success.

**Design:**

This study used a quantitative, correlational design.

**Methods:**

Retrospective analysis of records of 367 graduates over a 6‐year period tested the relationship of pass rate on the licensing examination to six variables: overall pre‐admission grade point average, entrance assessment scores, interview scores, remediation, programme length and exit assessment using point‐biserial correlations, and chi‐square analysis and logistic regression analysis.

**Results:**

Overall pre‐admission grade point average, entrance assessment scores, interview scores and exit assessment scores were positively correlated with student success. Although remediation and programme length were not correlated with success, 87% of the students participated in remediation. Most students (95%) successfully passed the Registered Nurse licence examination on their first attempt. While specific criteria were related to student success, further research is needed to determine the role of remediation.

## 1 INTRODUCTION

A fundamental alteration in educating nurses using non‐traditional pedagogies is needed to prepare future nurses for practice (Benner, Sutphen, Leonard, & Day, 2010). In the United States, nursing programme graduates are judged to be ready for nursing practice by passing the high stakes National Council Licensure Examination for Registered Nurses (NCLEX‐RN^®^) (Carrick, 2011; Cherkis & Rosciano, 2015; Langford & Young, 2013). Determining which admission criteria appropriately identify students who can be retained in the nursing programme and successful in the NCLEX‐RN is an ongoing challenge for faculty and administrators in nursing programmes (Cameron, Roxburgh, Taylor, & Lauder, 2011; Cherkis & Rosciano, 2015; McNelis et al., 2010; Timer & Clauson, 2011). Nurse educators, who implement admission policies and pedagogical practices, standardized testing and remediation efforts, could influence student progression and success performance on the NCLEX‐RN^®^ on the graduate's first attempt (Seidman, 2012; Simon, McGinniss, & Krauss, 2013). Nursing programmes commonly employ standardized assessments to identify at‐risk students unlikely to be successful in nursing programmes and on the NCLEX‐RN^®^ (Nibert & Morrison, 2013; Quinn, Smolinski, & Peters, 2018). The mastery learning approach (MLA) is a non‐traditional pedagogy, which posits that all students can achieve educational outcomes of any subject content if faculty provides appropriate and quality instruction through a variety of instructional strategies without regard to time needed to reach the outcome (Guskey, 1997; McGaghie, Issenberg, Cohen, Barsuk, & Wayne, 2011; Morgan, 2011). An essential component of MLA is remediation that incorporates corrective feedback to address individual learning difficulties and provides specific high‐quality corrective instruction (Guskey, 2010; McGaghie et al., 2011). A plethora of studies evaluated combinations of factors related to nursing student success although no studies were found using the combination of predictors of success in a MLA, Bachelor of Science in Nursing (BSN) programme. (Chen & Voyles, 2013; Cherkis & Rosciano, 2015; Shulruf, Wang, Zhao, & Baker, 2011).

## 2 BACKGROUND

To meet the needs of an increasingly diverse student population, an innovative private non‐profit university in the southwestern United States developed a curriculum based on the theoretical underpinnings of mastery learning. There are six points of MLA that are integral in the implementation of the educational philosophy including the classroom as a teacher, Block curriculum, active and collaborative learning, competency‐based education, assessment learning, early experiential learning and a classroom design that facilitates learning and interaction among students. The programme does not provide pre‐requisite courses. In the university, the college of nursing adhered to the university's pedagogy of integrated immersion through block scheduling (one specialty area content per block) to allow students to maximize comprehension and develop content mastery. High standards were set (90% pass on all assessments, laboratory skills and courses known as “blocks”), providing time and opportunities for faculty‐directed remediation and the use of ATI remediation instruments, so all students could achieve the same level of learning to be successful, but not all students succeeded.

Mastery learning research provided an increased opportunity for most students to reach the same level of achievement as the average students from a conventional class performing at the 70th percentile and students were found to have developed more positive attitudes towards learning and improve retention of content (Kulik, Kulik, & Bangert‐Drowns, 1990; Morgan, 2011; Shafie, Shahdan, & Liew, 2010). The implementation of mastery learning in nursing education is limited, although multiple studies revealed positive findings on the effectiveness of mastery learning programmes (Davis & Sorrell, 1995; McGaghie et al., 2011; Morgan, 2011). Few studies investigated strategies to improve the success of students who are at risk for failure (Carrick, 2011; Cherkis & Rosciano, 2015).

While no research was found addressing mastery learning and the role of remediation in a BSN programme, literature suggested that remediation might aid student success (Harding, 2012; Shafie et al., 2010). The implementation of mastery learning in medical and nursing education is limited mostly to simulation and clinical skill requirements, although studies in other areas of education have been conducted, revealing positive findings on the effectiveness of mastery learning programmes on both cognitive and affective educational outcomes (Barsuk, McGaghie, Cohen, Balachandran, & Wayne, 2009; Davis & Sorrell, 1995; McGaghie et al., 2011; Morgan, 2011).

Nursing educators have desired to admit qualified students and identify at‐risk students through admission criteria as predictors of success. Acceptance into the MLA nursing programme was based on GPA scores, a standardized entrance assessment score ([TEAS^®^] shown to assist faculty to identify potentially successful candidates) and an interview score (ATI, 2011; Wolkowitz & Kelley, 2010). The nursing faculty selected the Assessment Technologies Institute (ATI) Inc. to enhance their curriculum and assist students to comprehend the requirements needed to pass high stake tests.

**Figure 1:**
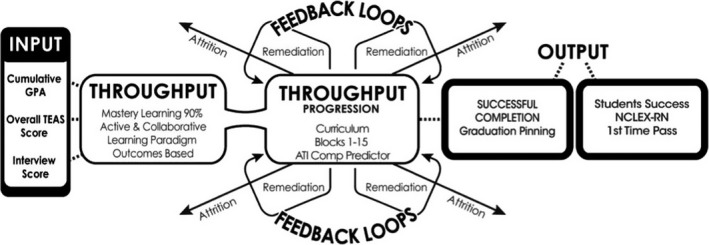
Original Marie Wright (2014) Model “Wright's system‐based theory of success in a MLA BSN programme”

This study used a system‐based framework (Figure 1) that included student admission criteria, student progression criteria including the programme's pedagogical model that reflects the university affiliate (von Bertalanffy, 1968; Carrick, 2011; Roseman University of Health Sciences College of Nursing, 2013; Simon et al., 2013; Wright, 2015). The model design provided an effective theoretical foundation for testing the relationships between the variables in this study.

The first system involves the input of the student's personal system achievements prior to entrance to the CON programme: cumulative pre‐admission GPA of required pre‐requisite courses, ATI TEAS score and the pre‐admission interview score. Throughput is the interpersonal system that includes the interaction of students, faculty and the pedagogical practices designed for student success, block immersion, mastery learning models that includes the ATI Comprehensive Assessment and Review Predictor (CARP) in the last block course and involves remediation throughout the blocks to assist students to master content and progress in their learning. Finally, the output criterion involves the successful graduation, length of time to complete the programme and first‐time NCLEX‐RN licensure success for all students. Education involves the use of many combinations of interventions needed to undertake a multitude of factors that may influence transform and maintain changes in personal and interpersonal systems and the social system (Simon et al., 2013).

Student input variables included grade point average (GPA), entrance assessment scores (TEAS^®^) Assessment Technologies Institute (ATI, 2011) and interview scores. Study findings suggested that variables of student input and overall GPA on admission are the best predictors of success outcomes (Simon et al., 2013). Pre‐admission GPA remains the only consistent predictor of success from the multiple studies conducted to identify predictors of student success (Grossbach & Kuncel, 2011; Simon et al., 2013; Timer & Clauson, 2011). Similarly, the ATI TEAS^®^ pre‐admission standardized assessment scores have shown a high correlation with successful students (ATI, 2011; Cherkis & Rosciano, 2015; Horton, Pollek, & Hardie, 2012; Wolkowitz & Kelley, 2010), while pre‐admission interview and other student input criteria were not strong indicators of success (McNelis et al., 2010). Studies have shown that students who are older and have a higher re‐requisite GPA have a greater likelihood of successful completion of a nursing programme (Kowitlawakul, Brenkus, & Dugan, 2013; Pryjmachuk, Easton, & Littlewood, 2009). Studies of multiple combinations of variables have not isolated those traits that consistently identify the students most likely to be successful, indicating the need for further research.

The non‐academic admission interview has been used in higher education as a means to determine applicant's career decision‐making process and determine non‐cognitive factors recognized from the applicants' understanding of the commitment and lifestyle changes necessary for success in the programme. Identification of individual characteristics, such as motivation, caring and compassion, integrity, interpersonal skills, altruism and respect, is crucial to the student selection process and ultimately forms the essential qualities of the professional nurse (Ehrenfeld & Tabak, 2000; McBurney & Carty, 2009; Perkins, Burton, Dray, & Elcock, 2012; Rosenberg, Perraud, & Willis, 2007). Faculty designed an interview instrument based on the American Nurses Association Code of Ethics for Nurses (ANA, 2015) to measure applicants' motivation, team skills, problem‐solving skills, caring/compassion, professionalism, leadership and communication.

The throughput criteria (Simon et al., 2013) included the number of remediation attempts, programme length and ATI (CARP) (ATI, 2013) exit assessment score. Remediation, which is integral to the mastery learning philosophy, includes corrective feedback and procedures that address individual learning difficulties and specific remediation activities that are considered high‐quality corrective instruction (Guskey, 2010). The remediation system allowed all students one more opportunity to take a second similar assessment to the one not passed with a score of 90% or above to move forward in the programme. If the remediation assessment was not passed, the student received a grade of No Pass and had the opportunity to repeat the Block (Roseman University of Health Sciences College of Nursing, 2013). Few studies examined the remediation interventions that assist students with low grades to achieve success on NCLEX‐RN^®^ (Simon et. al., 2013).

The second throughput variable was programme length, which was measured by the number of months between admission to the first nursing course and completion of the last nursing course, an expected period of 18 months. Studies on nursing programme length's influence on student success are limited. The third throughput variable was the score on the ATI (CARP). The faculty used the ATI (CARP) to identify students' need for further remediation prior to graduation. The ATI (CARP) has construct validity and evidence exists that students who score at level 2 or above were likely to successfully pass the NCLEX‐RN^®^ (Liu & Mills, 2017; Morahan, 2011; Sims, 2012). Remediation based on ATI (CARP) has shown positive results to promote student success on first‐time NCLEX‐RN^®^pass rates (Horton et al., 2012).

The purpose of this study was to investigate the relationship between the input variables and throughput and feedback loop variables and student success. Input variables were the overall GPA score, overall TEAS^®^ score and overall interview score. Throughput and feedback loop variables were the number of times student remediated, length of time to complete the mastery learning Bachelor of Nursing programme and ATI (CARP) score of 90% or greater probability. The student success outcome was passing NCLEX‐RN^®^on 1st attempt.

## 3 METHOD

Using a retrospective, quantitative, correlational predictive design, this study investigated the relationship of admission criteria and programme strategies to the outcome measure of success.

The data for the following variables were collected from existing student records:

1. Overall pre‐admission GPA score was the calculated GPA for all previous college level course work accepted by the university.
2. Overall TEAS^®^ entrance assessment score was calculated by ATI and is the student's overall TEAS^®^standardized assessment score inclusive of the Reading, Math, Science and English aptitude scores.
3. Overall pre‐admission interview score for oral and written communication was the average of two individual interviewer scores. The interview questions and score sheet and the academic achievement score sheet were used in the decision process of admission to the nursing programme. The score sheet mirrored the student's information from the archive records. The data collected for each student were the same, which validated the collection instrument.
4. Number of times student remediated assessments or blocks by reviewing concepts from course where the student did not score at least 90% on the summative evaluation.
5. Length of time in months that was necessary to complete the mastery learning BSN programme from the first date of attendance.
6. Overall ATI (CARP) standardized exit assessment score of 69.3 or ≥90% probability of passing NCLEX‐RN on the first attempt. Students could take the standardized exit assessment instrument up to three times, so for students who took the assessment more than once, the standardized exit assessment instrument score was measured as the average of each standardized exit assessment instrument score. Student scores from the ATI (CARP) assessments were compared with ATI's assessment forms for the year used. The 2010 Forms A and B listed the score students needed to achieve to predict the probability of passing the NCLEX‐RN^®^. The faculty determined that 90% for all assessments must be obtained to achieve mastery. In Block 15, the ATI (CARP) was given as an assessment in the block. Students must pass the ATI CARP assessment with the predicted probability score that equalled a 90% likelihood of passing the NCLEX‐RN^®^on the first try and is correlated with the year the assessment was given. Students must achieve a 69.35 score or above to pass Block 15.
7. The Pass/Fail grade on the NCLEX‐RN^®^ on 1st attempt. The NCLEX‐RN^®^, established by The National Council of State Boards of Nursing, uses a decision consistency statistic to determine reliability of the NCLEX‐RN^®^ (NCSBN, 2013).

Institutional review boards of the university where data were collected, and the primary investigator's university approved the study. Family Educational Rights and Privacy Act guidelines regarding student records were followed. Student records were de‐identified before data were collected. Data were imported into Statistical Package for the Social Sciences software (SPSS version 20.0) for analysis.

### 3.1 Population and sample

The population consisted of retrospective records of all nursing students (*N* = 504) admitted to the nursing programme on two both campuses in 2008 through 2012. When the data were imported for analysis, seven students had not taken the NCLEX‐RN^®^ examination before data collection and were not included in the study. The final sample included records of (*N* = 374) students who graduated and received a score for writing the NCLEX‐RN for the first time.

### 3.2 Analysis

Frequencies and percentages were calculated for gender, race, remediation status, admission year and pass on NCLEX‐RN^®^on the first attempt. Means and standard deviations of the students' overall pre‐admission GPA, standardized entrance assessment score, overall interview score, age, length of time of complete the BSN programme in months, ATI (CARP) scores on first and second attempts were included in the descriptive statistics. All student input and throughput variables were assessed for correlation with the NCLEX‐RN^®^first‐time pass/fail variable. Point‐biserial correlations were used to determine whether there was a significant relationship between whether a student passed the NCLEX‐RN^®^on the first attempt and overall pre‐admission GPA, standardized entrance assessment score, overall interview score, length of time of complete the BSN programme and standardized exit assessment instrument score. The assumption of normality was assessed using a Kolmogorov–Smirnov (KS) test. Cohen's standard (Cohen, 1988) was used to evaluate the correlation coefficient to determine the strength of the relationship, where coefficients between 0.10–0.29 represent a small association; coefficients between 0.30–0.49 represent a medium association; and coefficients above 0.50 represent a large association or relationship.

A binary logistic regression was conducted to determine the extent to which length of time to complete BSN, pre‐admission GPAs, standardized entrance assessment instrument scores, overall interview scores, remediation status and standardized exit assessment instrument scores influenced a pass on the NCLEX‐RN^®^first attempt. This analysis viewed all variables as they relate to NCLEX‐RN^®^success in tandem so that each variable's individual effect could be examined while controlling for the effects of each other variable. In addition, this analysis allowed the researcher to view each of these effects while controlling for factors such as gender (male, female), age (continuous) and ethnicity (Hispanic, American Indian/Alaskan Native, African American/Black, White, Pacific Islander, Asian, Indian, Middle Eastern and other).

## 4 RESULTS

### 4.1 Demographics

The 367 students who graduated from the programme either participated in remedial classes (319, 87%) or did not (48, 13%) over a 6‐year period from 2008 through to the 2012 admission cohort graduating in 2013. Students who did not achieve the benchmark on the first try on the ATI (CARP) were allowed a second attempt and a third if they did not reach the benchmark by the second. First ATI (CARP) attempts averaged to 77.11 (*SD* 6.62), while second attempts averaged at 78.49 (*SD* 7.27) and third attempts were 79.95 on average (*SD* 7.42).

### 4.2 Variables

The GPA on pre‐requisite courses ranged from 2.75–4.0 with an average of 3.34 (*SD* 0.27). Scores on the standardized entrance assessment ranged from 63.5–94 averaging 79.87 (*SD* 5.63). Scores on the pre‐admission interview ranged from 22.5–61.8 averaging 43.61 (*SD* 4.12). 87% participated in remediation, which was available to all students; 13% did not. Average time to graduation was 19.36 months ranging from 18–39 months (*SD* 2.93). Of the 367 graduates, 349 (95%) passed the NCLEX‐RN^®^on the first attempt.

### 4.3 Hypothesis testing

A significant weak relationship existed between overall pre‐admission GPA scores (rpb = 0.18, *p* < 0.001), overall standardized entrance assessment score (rpb = 0.14, *p* = 0.015), overall interview score (rpb = 0.13, *p* = 0.014) and standardized exit assessment instrument score (rpb = 0.19, *p* < 0.001) with the pass on the first NCLEX‐RN^®^attempt. There was no significant relationship between the students who remediated and passed NCLEX‐RN^®^on the first attempt (χ^2^(1) = 2.85, *p* = 0.091). No significant relationship existed between length of time to completion and a pass on the first NCLEX‐RN^®^attempt (rpb = −0.02, *p* < 0.707). Students with scores on the standardized entrance assessment scores below 70 did not follow the trend of those who scored above 70, wherein higher scores on the standardized entrance assessment corresponded with higher passing rates; thus, these students' were assessed independently. A total of 11 students scored below 70 on the standardized entrance assessment, and these students were examined for the length of time to completion of the programme, as well as pre‐requisite GPAs and the number of remediation attempts. Two students did not participate in remediation. The remaining students participated in remediation. A problem related to the testing process may have caused the student to score under 70 on the standardized entrance assessment but did not influence overall success for the student.

For the binary logistic regression, results of the overall model fit indicated a significant model (χ^2^ (10) = 22.08, *p* = 0.015, Nagelkerke *R* ^2^ = 0.32). The standardized entrance assessment instrument scores (Wald = 4.06, *p* = 0.044) and the standardized exit assessment instrument scores (Wald = 4.26, *p* = 0.039) both had a predictive relationship with NCLEX‐RN^®^pass rates that were significant beyond the influence of any other variables. Examination of the odds ratios determined that for every unit increase in the standardized entrance instrument scores, participants increased odds of passing the NCLEX‐RN^®^on the first attempt by a factor of 1.18. Similarly, for every unit increase in standardized exit assessment instrument scores, participants increased odds of passing the NCLEX‐RN^®^on the first attempt by a factor of 1.20.

## 5 DISCUSSION

In agreement with findings of previous studies (Grossbach & Kuncel, 2011; Simon et al., 2013; Timer & Clauson, 2011), the results indicated that pre‐admission GPA scores were a predictor of student success. However, remediation in the mastery learning BSN programme may have offset the requirement for a high pre‐admission GPA to be admitted to the programme because students requiring multiple student remediation assessments still did well with a NCLEX‐RN^®^pass rate of 88%. Students from this mastery learning BSN programme who scored higher on the standardized entrance assessment instrument had higher likelihood of passing on the NCLEX‐RN^®^on the first attempt. In addition, remediation may have been a factor in the success of the 11 students who scored below 70 on the standardized entrance assessment instrument and were successful on NCLEX‐RN^®^the first time. This study provided evidence that pre‐admission interviews can assess the personal characteristics that may predict student success in a mastery learning BSN programme, supporting findings from a recent study of dental hygiene students reporting that pre‐admission interviews may be an important consideration when evaluating student retention (Sanderson & Lorentzen, 2015).

Insufficient evidence existed to support that remediation was related to passing the NCLEX‐RN^®^on the first attempt. However, remediation is a key component of success in a mastery learning programme. Nine of the eleven students participated in several remediation attempts and were successful on the first attempt of the NCLEX‐RN^®^licensure indicating that, despite the findings of previous researchers and the insignificant findings in this study, the academic value of remediation remains as a variable that improves student success. While the results of the analysis indicated that there was not significant relationship between length of time to completion and passing NCLEX‐RN^®^on the first attempt, six of the eleven students took longer to complete the BSN programme and were successful on the first attempt of the NCLEX‐RN^®^licensure, indicating that the length of programme time potentially improves student success. Although the findings indicated that the number of remediation attempts was not related to success, 87% of students participated in remediation. Students who successfully completed the programme regardless of the number of times they remediated or the length of time that it took to complete the programme may have the same chance of achievement as their peers who did not extend the length of the programme by remediating. Overall results of the student outcome revealed that most students (*N* = 342, 95%) had successful output success on NCLEX‐RN^®^on the first attempt.

### 5.1 Limitations

Limitations included using a non‐randomized sample with data from one BSN programme. Bias introduced during the interview process and reflected in the pre‐admission interview scores may have influenced the results. Lack of knowledge of the institution's influences on each student's educational background and grades attained on pre‐requisite courses was a limitation. Data did not include knowledge and/or variables that influenced each individual student's life experiences before enrolling as a nursing student. Information about study habits and success in previous educational endeavours was not available and may have relevance to the outcome of the study. Students in the sample may have taken different versions of the NCLEX‐RN^®^test plan. Students who graduated before April 2013 took the 2007 NCLEX‐RN^®^test plan. Students, who graduated after April 2013, took the 2010 NCLEX‐RN^®^test plan. Each time a new NCLEX‐RN^®^test plan was implemented; there was a slight drop in the pass rate results initially after the new standard is introduced (NSBN, 2017).

## 6 SUMMARY

The purpose of this study, with a correlation prediction design, was to determine whether a relationship existed between the student predictor variables and the student outcome success of passing the NCLEX‐RN^®^on first attempt. Results of the study demonstrated that GPA scores, standardized entrance assessment instrument score, interview score and a standardize exit assessment score are positively correlated with student success as measured by the graduate passing the NCLEX‐RN^®^on the first attempt in a non‐traditional mastery learning BSN programme. Although the findings indicated that remediation and a longer time in the programme did not show a significant correlation to student success, these throughput variables may be facilitators of learning. Results of the outcome variable of student success revealed that most students (*N* = 342, 95%) had successful output success on first‐time NCLEX‐RN^®^licensure.

Mastery learning is a non‐traditional pedagogy that demands further investigation. Future research may discover relationships between the MLA of expectations and other measures of student success. Further study of how mastery learning opportunities nursing BSN students may help improve academic achievement of students from diverse backgrounds who have lower pre‐admission GPA scores. Further investigation is needed to understand how remediation and increased time allows students with limited resources to develop positive attitudes towards learning and retention of content. Future research into the subtests (Reading, Math, Science and English language use) of the standardized ATI entrance assessment instrument could yield a better way to identify potentially successful students and provide insights into the type of remediation required by individual students early in the nursing programme. Comparing students who do and do not remediate is another area that requires further study. Future research could investigate internationally trained nurses' use of the virtual ATI for NCLEX success. Also, further examination of diverse student populations and those for whom English is a second language benefit from ATI training and assessment tools. While there is more to learn about the factors that support student success, the overall findings from this study add to the body of knowledge on mastery learning in a BSN programme. The findings indicated that a BSN programme that used a mastery learning, block immersion approach and considered their pre‐admission GPA, ATI TEAS^®^ assessment scores, admission interviews and ATI (CARP) exit assessment scores graduated students with a high level of success on the graduates' first attempt on the NCLEX‐RN^®^licensing examination.

## CONFLICTS OF INTEREST

No conflict of interest has been declared by the author(s).

## AUTHOR CONTRIBUTIONS

Marie Rolf: Principal Investigator (PI) and data collector of the parent study. All authors contributed to the study conceptualization, interpretation of findings, and manuscript preparation and revision. All authors agreed to be accountable for all aspects of the work to ensure that questions related to the accuracy or integrity of any part of the work are appropriately investigated and resolved.
